# Ocular Manifestations of Ectrodactyly-Ectodermal Dysplasia-Cleft Palate (EEC) Syndrome: A Case Report

**DOI:** 10.7759/cureus.36086

**Published:** 2023-03-13

**Authors:** Mohammad Fahim Mohd Jais, Tai Wan Dien, Wen-Jeat Ang, Raja Norliza Raja Omar, Nor Fadhilah Mohamad

**Affiliations:** 1 Ophthalmology, University of Malaya Eye Research Centre, Kuala Lumpur, MYS; 2 Ophthalmology, Hospital Melaka, Melaka, MYS; 3 Ophthalmology, Hospital Shah Alam, Kuala Lumpur, MYS; 4 Ophthalmology, Hospital Universiti Sains Malaysia, Kelantan, MYS; 5 Ophthalmology, University Malaya Medical Centre, Kuala Lumpur, MYS

**Keywords:** ectodermal dysplasia, ectrodactyly, cornea, cleft-palate, eec syndrome

## Abstract

Ocular manifestations are common associations of ectrodactyly-Ectodermal dysplasia-cleft palate (EEC) syndrome. We would like to report a case of a 48-year-old patient with EEC syndrome who manifested ocular and extraocular signs and symptoms. The ophthalmic findings in this patient included chronic blepharitis and absence of meibomian gland. There was also a presence of hazy cornea with vascularized corneal stroma and symblepharon involving the lower lid. Systemic conditions showed generalized dry and scaly skin with hand-foot split deformity. Therefore, ophthalmologists should be alert to spot and diagnose this condition as prompt treatment should be commenced considering this can be sight-threatening.

## Introduction

The ectrodactyly-ectodermal dysplasia-cleft palate (EEC) syndrome is a rare form of ectodermal dysplasia and autosomal dominant disorder inherited as a genetic trait that has variable penetrance and expression within one family. Most of the cases are associated with mutation of the TP63 gene and it affects males and females in equal number [1]. It is characterized by the triad of ectrodactyly, ectodermal dysplasia, and facial cleft.

Ectrodactyly is a condition characterized by deficiency or absence of one or more fingers or toes and is also known as split hand-split foot deformity. Severity may vary as it could affect all four hands and feet or only cause mild malformations. Clefting of the lip or palate is another character of EEC syndrome. Affected individuals also might have maxillary hypoplasia, a broad nasal tip, and choanal atresia. The severity of ectodermal dysplasia is also highly variable but it always affects skin, sweat glands, teeth, and hair. The patient might present with dry and itchy skin, coarse scalp hair, sparse eyebrow and eyelashes, hypodontia, dysplastic nails, hypohidrosis, and dry mouth.

It was first reported in 1936 by Cockayne and subsequently by Walker and Clodius in 1963 where they described a patient with cleft lip and palate with lobster-claw deformities [2,3]. The term EEC syndrome was suggested by Rudiger et al. in 1970 where they elaborated the clinical findings with ectodermal dysplasia [4]. EEC syndrome has been reported in many case reports but only a few in literature and publications described ocular manifestations.

This syndrome is rare and relatively unknown even among ophthalmologists, although it can lead to severe visual symptoms and eventually lead to visual impairment. This condition will not be noticed in the early years and if, inadequately managed, it may be too late later in life and the patient will be visually handicapped due to untreated ocular manifestations and involvement. 

This article was previously presented as e-poster at the 11th Conjoint Ophthalmology Scientific Conference University Malaya- Asia Pacific Ophthalmic Trauma Society (COSC UM-APOTS) Ophthalmic Trauma Meeting 2022 on September 17, 2022.

## Case presentation

**Figure 1:**
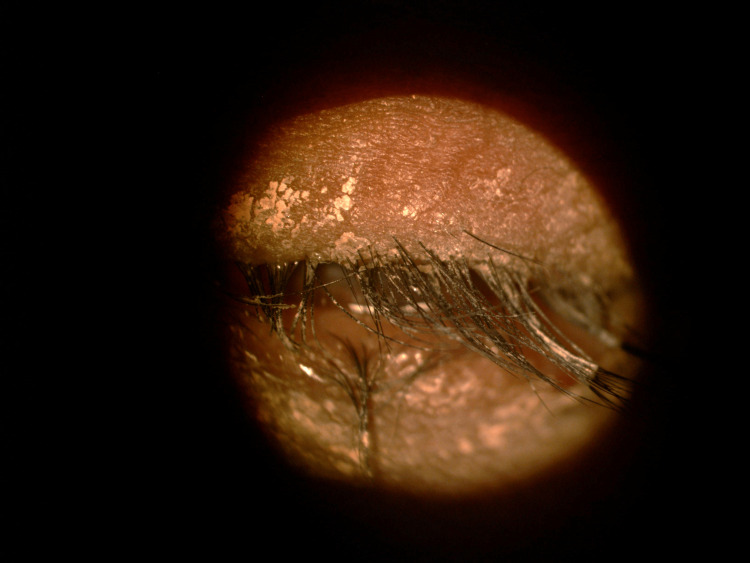
Chronic blepharitis and matted eye lashes

**Figure 2:**
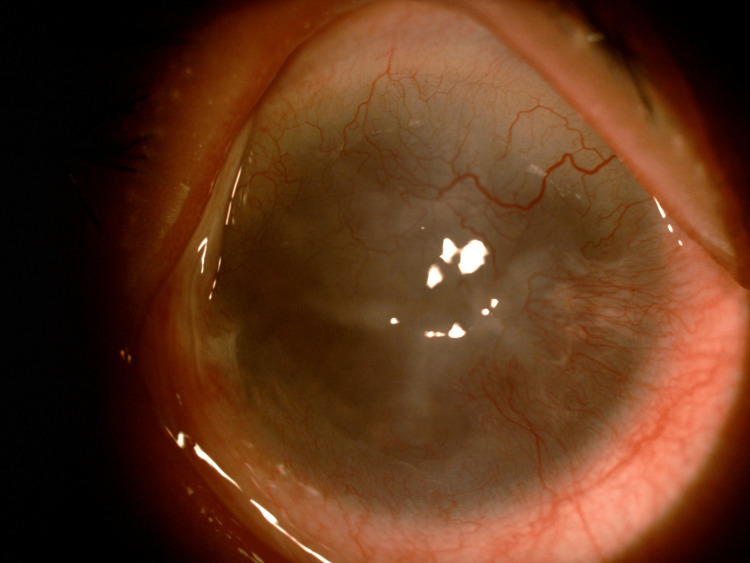
Hazy cornea with extensive stromal vascularization

**Figure 3:**
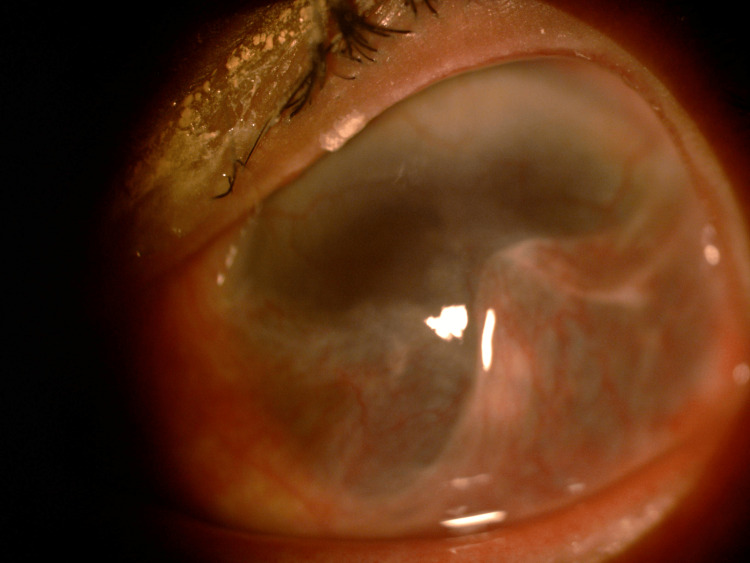
Symblepharon over the left eye

**Figure 4:**
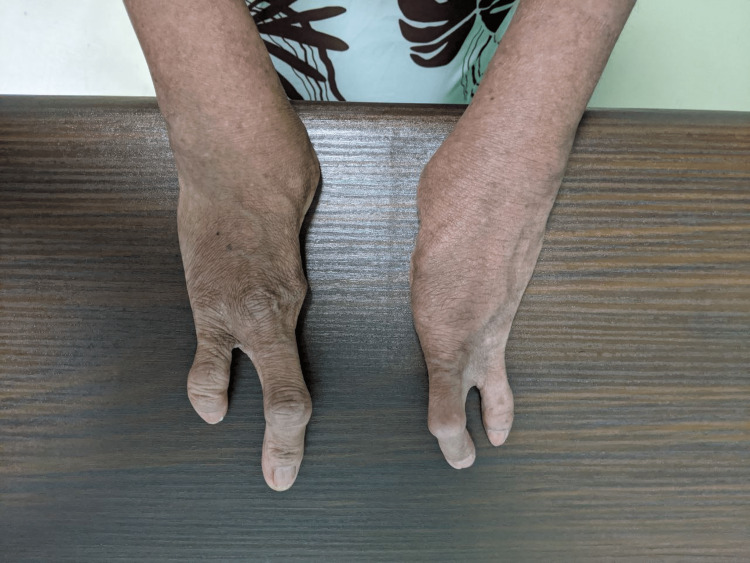
Lobster-claw hands

**Figure 5:**
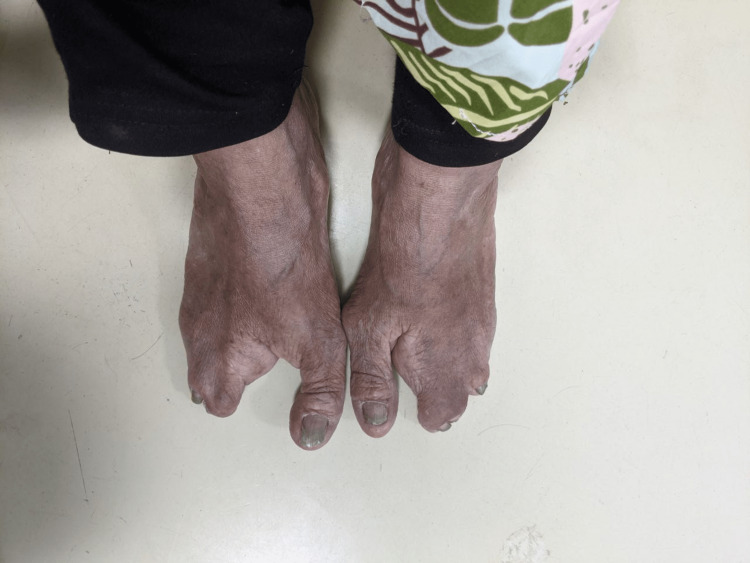
Split-foot deformity

**Figure 6:**
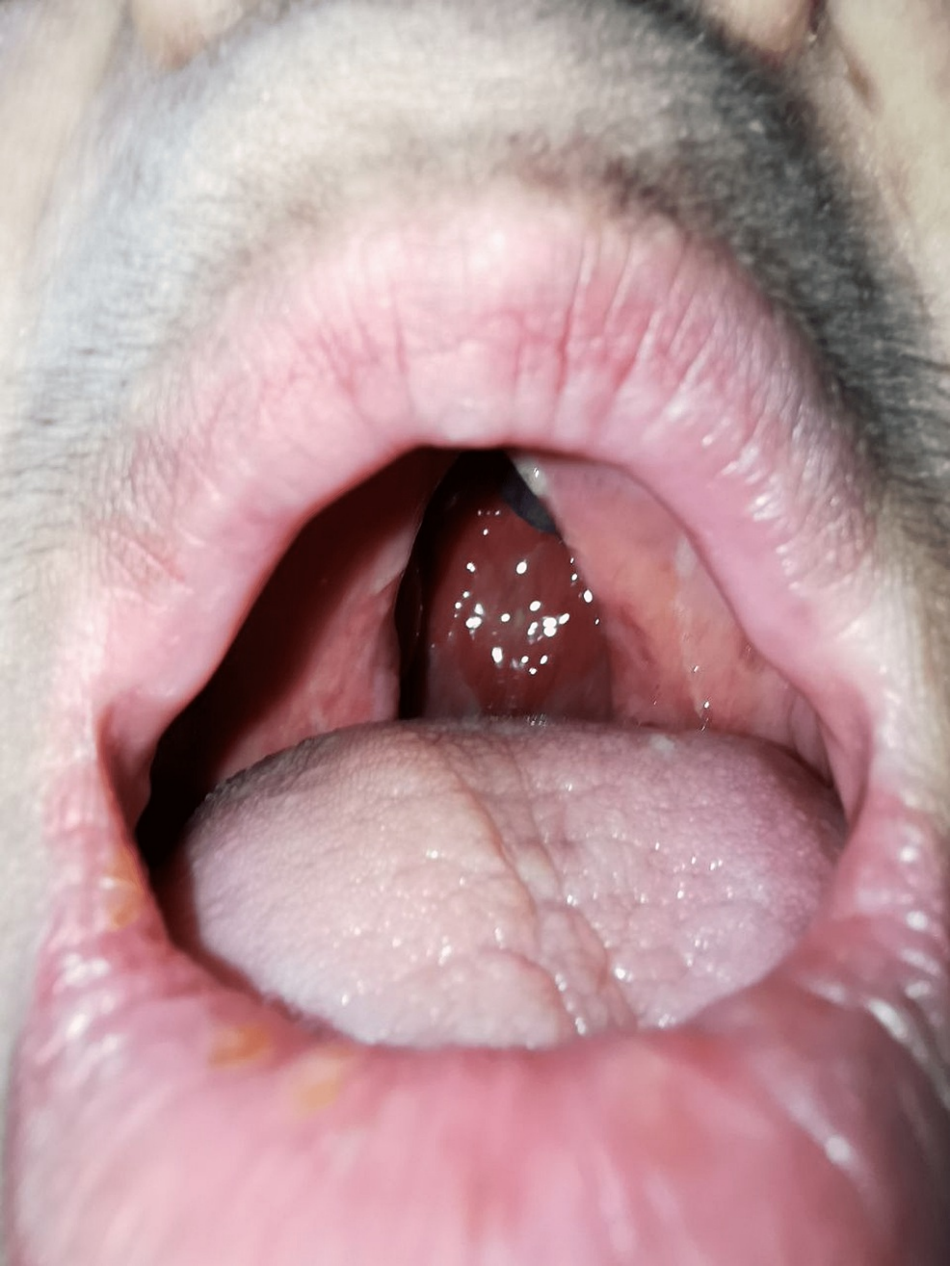
Cleft-palate deformity

A 48-year-old woman working as a janitor presented with reduced vision in both eyes for years associated with eye redness and photophobia. She had history of multiple visits and follow-ups to ophthalmology clinics but she was lost to subsequent follow up. She was not compliant to her topical eye drops due to difficulty of instilling eyedrops on her own. Eye examination showed both eyes’ visual acuity as 6/36, and no relative afferent pupillary defect was detected. There were chronic blepharitis over both eyes with matted lashes and scaly eyelids and absence of meibomian glands (Figure 1). The cornea showed obvious central opacity with 360 degrees deep stromal vascularization (Figure 2). Upon staining the cornea with fluorescein strip, there were generalized punctate epithelial erosions with no epithelial defect. The left eye showed inferotemporal symblepharon (Figure 3). Anterior chamber view was hazy due to cornea opacity and there was no view of the fundus. B-scan showed clear vitreous and the retina was flat. Systemic examination showed she had generalized dry skin, both hands and feet split deformity (lobster-claw hand), maxillary hypoplasia, and cleft palate which caused her difficulty in her speech (Figure 4-6) .

Initially, the patient was suspected of having connective tissue disease. However, all relevant investigations were negative. There was no further molecular or genetic testing done. Patient was married and had no children. There are no other known family members with similar condition.

Patient was started with extensive eye lubricants to improve her symptoms and to prevent ocular complication such as corneal ulcer. Long-term plan such as removal of symblepharon and bandage contact lens were offered but she was undecided. Patient was referred to plastic surgery team for her cleft palate. The next review showed improvement of corneal punctate epithelial erosions and reduced conjunctival redness. Patient was scheduled for subsequent follow-ups and monitoring. However, she was lost to follow-up and was uncontactable.

## Discussion

There are various ocular manifestations and severities seen in EEC syndrome ranging from severe dry eyes due to lacrimal and meibomian gland dysfunction, recurrent dacryocystitis, chronic blepharitis, cornea vascularization, and deep cornea scarring and ulceration. All these manifestations are due to two main changes in the eye and its adnexa, atresia of the lacrimal duct and absence of the meibomian gland, which lead to full-blown ocular surface disease.

Extraocular symptoms such as cleft palate and limb abnormalities always get surgically repaired in early life and the patient will be able to rehabilitate well. However, ocular complications will manifest later in life and the patient will exhibit irreversible severe keratopathy, thus leading to functional disability and blindness.

There are multiple theories of corneal pathologies in EEC syndrome, and one of them is tear-film instability as described by Wilson et al. in the 1970s [5]. They suggested that the defect of the tear film and malnutrition of the cornea will cause the formation of vascularized scars. This condition is worsened by epithelial defect due to generalized ectodermal dysplasia and limbal stem cells deficiency as described by Di Iorio et al. due to mutation of the P63 gene [6,7]. A case was reported in 1984 by Mondino et al. of a 27-year-old female patient who had a reduction in conjunctiva goblet cells causing mucus deficiency in the tears leading to tear-film instability [8]. The patient also had a total absence of the meibomian gland in both upper and lower lids, which was confirmed by full-thickness biopsy. Lack of lipid component contributed to tear-film instability in this patient. In Japan, an EEC syndrome patient with tear-film instability was treated with low-dose lipid application for the ocular surface and showed promising results by alleviating the symptoms and resulting in a positive outcome [9].

Another contributing factor is a defective lacrimal drainage system, which could lead to persistent infection and corneal alteration. Patients can be as young as infants as reported in the study by Käsmann B et al. in 1997 of a father and his son who had EEC syndrome manifested by typical extraocular manifestations [10]. The 18-month-old child was first brought to the ophthalmology department for constant epiphora and recurrent conjunctivitis, which later on revealed there was nasolacrimal duct atresia. The treatment of such cases is not always straightforward and lacrimal surgery is the treatment of choice. However, there was a case where the patient’s ocular condition failed to improve and ulcerated despite bilateral dacryocystorhinostomy and dacryocystectomy [11].

In severe cornea involvement, a patient might come with spontaneous corneal perforation. There are multiple reported cases pertaining to this complication. In 1989, McNaab et al. reported two cases of spontaneous corneal perforation due to corneal ulceration which was treated with a bandage contact lens and medical management [12]. Another reported case by Felipe et al. in 2012 described a three-year-old child with spontaneous corneal perforation, which required emergency examination under anesthesia [13]. They noted the presence of descemetocele with Seidel’s positive. The case was managed by medical treatment, corneal glue, bandage contact lens, and surgery.

## Conclusions

In conclusion, late diagnosis and inappropriate therapy will cost patients their vision. Early management of this syndrome is crucial because it is difficult to maintain the ocular surface integrity when there is limbal stem-cell deficiency and other associated factors such as chronic blepharitis, meibomian gland aplasia, lacrimal drainage dysfunction, and toxicity from the medication. Monitoring the patients during follow-up at frequent intervals is also vital to detect early progression of the disease.
